# Real Neurolaw in the Netherlands: The Role of the Developing Brain in the New Adolescent Criminal Law

**DOI:** 10.3389/fpsyg.2020.01762

**Published:** 2020-07-29

**Authors:** Stephan Schleim

**Affiliations:** Theory and History of Psychology, Heymans Institute for Psychological Research, Faculty of Behavioral and Social Sciences, University of Groningen, Groningen, Netherlands

**Keywords:** neurolaw, legal responsibility, adolescent criminal law, forensic psychiatry, neuroethics

## Abstract

Previous publications discussed the conditions under which courts admitted or could admit neurotechnological evidence like brain scans. There were also first attempts to investigate legal decisions neuroscientifically. The present paper analyzes a different way in which neuroscience already influenced the law: The legal justification of the new Dutch adolescent criminal law explicitly mentions findings on brain development to justify a higher maximum age for the application of juvenile criminal law than before. The lawmaker’s reasoning is compared with the neuroscientific studies on which it is based. In particular, three neurodevelopmental publications quoted by the Dutch Council for the Administration of Criminal Justice and Protection of Juveniles to justify that adolescents can be legally less responsible are analyzed in detail. The paper also addresses possibilities under which brain research could improve legal decision-making in the future. One important aspect turns out to be that neuroscience should not only matter on the level of justification, but also provide better instruments on the individual level of application.

## Introduction

*Neuroethics* has been introduced as a twofold endeavor: the ethics of neuroscience and the neuroscience of ethics. The former could be understood as a variation of bio- or medical ethics, addressing ethical issues involving neurotechnology and the brain (Farah, 2012), the latter as an extension of moral psychology with instruments like the electroencephalogram, functional MRI (fMRI), or transcranial magnetic stimulation (Schleim, 2008). Neuroethics has now been institutionalized with its own journals, trainings, and associations (Leefmann et al., 2016). *Neurolaw* could be understood in a similar vein: legal regulation of neurotechnology on the one hand (Goodenough and Tucker, 2010; Spranger, 2012) and the neuroscience of legal decision-making on the other (e.g., Schleim et al., 2011). It is yet less institutionalized than neuroethics, but recently in this journal, Bigenwald and Chambon (2019) discussed the possible contribution of neuroscience to the law and concluded that neurotechnology might primarily improve the fact-finding in court. I am demonstrating in this paper that, beyond such theoretical considerations (Greene and Cohen, 2004), the Netherlands provide an example of real neurolaw in the sense that the lawmakers essentially justified the new adolescent criminal law in effect since 2014 with neuroscientific studies. This might actually be understood as a third branch of neurolaw: How can and does neuroscience change the law?

This is not just an abstract debate, as criminal court sentences involving adolescent offenders have already been and are being affected: For example, more criminals are sent to juvenile prisons (Schmidt et al., 2020). The analysis of the legal justification behind this is particularly relevant to theoretical psychology, too, as the concepts of responsibility and responsible behavior and their related cognitive and emotional processes are an essential mediator between neuroscience and the law in this area. After investigating the justification of the Dutch legal initiative in detail, I will discuss some more general aspects concerning the utility of neuroscience for the law and conclude the paper with an outlook for the future and possible implications for other legal domains.

## Neuroscience in the Legal Justification

Countries differ in how they treat adolescent—meaning the transition period from child to adulthood—offenders. Llamas and Marinaro (2020) recently provided an overview of Latin America, where the minimum age of holding offenders criminally responsible differs between 12 and 16 years and the maximum penalty given to adolescents varies between 3 (e.g., Brazil) and 15 (e.g., Costa Rica) years. In Netherlands, where the case of the present paper takes place, a law becoming effective in 1965 introduced the possibility to sentence 18- to 20-year-old offenders according to the rules for juvenile offenders, aged 12 to 17 years (Barendregt and van der Laan, 2019). These rules emphasize *pedagogical* goals over *punitive* aims. With the law in effect since April 1, 2014, the maximum age for this has been increased to 22 years^1^, while at the same time increasing the maximum sentence and introducing new possibilities for indefinite detention for the most serious adolescent offenders (Schmidt et al., 2020). Relevant for the purpose of the present paper is the justification of the new law, which I will summarize next.

In 2010, the newly formed government of Netherlands decided to address the problem of adolescent offenders more seriously. The following year, the State Secretary of Security and Justice presented a proposal, arguing that 15- to 23-year-olds committed 30% of the offenses. The State Secretary wrote:

“Research shows that many psychological functions which are important for the formation of socially desired behavior come to a full development only after the 20th year of age. This concerns, among others, the inhibition of impulses, the realization and consideration of long-term consequences, the regulation of emotions, and the development of empathic capacities. Considering the fact that these functions are not yet completely developed in adolescents, rule-breaking behavior and criminality occur relatively frequent in adolescents.”^2^

This suggests a causal link between the developmental stage of the said psychological faculties and offensive behavior. Another year later, the State Secretary presented the proposal for the new adolescent criminal law, including the following justification, where neuroscience and the brain enter the stage:

“The recent scientific findings related to the development of important brain functions during adolescence lend support for the intention to reach an independent treatment of adolescents. These insights come from developmental psychology and are confirmed by more recent neurobiological research. They are briefly summarized by the Council for the Administration of Criminal Justice and Protection of Juveniles with proposals for introducing a youth criminal law. That particular risk behavior occurs between the age of 15 and 23 can also be attributed to an incomplete development of important brain functions. The core of what science teaches about this is that the psychological development of adolescents does not stop when they reach 18 years of age and that essential developments only occur thereafter. The still incomplete emotional, social, moral, and intellectual development is a partial cause of the situation that a big part of (youth) criminality occurs during adolescence, but also ends before the 23rd year of age. […] Modern research on the functioning of the brain aided by scanning techniques is said to explain that adolescents let themselves be guided more by brain parts reacting to immediate reward than adults.”^3^

Besides the psychological faculties previously mentioned (like inhibition of impulses or emotion regulation), the justification here also refers to an increased susceptibility to peer pressure and lack of autonomy. We thus see that the lawmaker implies an even stronger link between brain development and offensive behavior than before. The quoted portion contains all (four) references to the brain of the justification of the new law^4^. To better understand the science behind this argument, I shall finally quote part of the proposal by the said Council, from the section “The psychological and biological development of the young”:

“Thus the growth of the young continues approximately until the 16th to 18th year. The gray matter of the brain develops earlier: An increased growth of the brain is reported until 14 to 15 years of age. Just around the 25th year prefrontal cortex functions such as planning and flexibility are completely grown. […] This means that specific risk behavior often occurring among adolescents between the 15th and 23rd year is partially caused by the incomplete development of particular important brain functions. The most essential development with respect to these brain functions only occurs after the 20th year. […] Research on the functioning of the brain using scanning techniques shows that adolescents let themselves often be controlled by a brain region reacting to immediate rewards, the *nucleus accumbens*, while the brain of persons older than 25 years show a stronger activation in the amygdala and prefrontal cortex. This lets the latter group consider the long-term consequences more in dangerous situations. […] Only when the prefrontal cortex has fully matured, the adolescents are better capable of regulating their emotions than before that time. […] It can be concluded that the development of adolescents usually is not completed until the 23rd year.”^5^

Before discussing the actual neuroscientific publications on which these claims are based in the next section, let us see the article of the Dutch Criminal Code as it was finally passed by the parliament and came into effect on April 1, 2014, based on the justification presented above:

“Regarding the young adult who, at the moment of committing the punishable act, has reached the age of 18, but not yet 23 years, the judge can judge according to articles 77 *g* to 77 hh, if he finds reasons for this in the personality of the offender or the circumstances in which the act is committed.”^6^

The further articles referred to here (77 *g* to 77 hh) are the—usually more mild and pedagogic—regulations for juvenile offenders, such as doing social work or serving the sentence in a juvenile prison. Procedural regulations not discussed here already allow a different placement before the trial and the transfer of the case to a dedicated youth court. More of these practical aspects have been discussed in earlier publications (e.g., Schmidt et al., 2020). It is interesting to note that the Dutch judges also relate to the brain in their public explanation of the new adolescent criminal law: “The thought is that as long as the brain is in development, the behavior of the suspect can be corrected maximally. Thus the chance of recidivism becomes as small as possible.”^7^ They praise the new regulations as allowing tailor-made solutions for individual offenders. Let us thus have a closer look at the neuroscientific evidence.

## Analyzing the Neuroscientific Studies

As we have seen in the previous section, the legal justification referred to a report by the Dutch Council for the Administration of Criminal Justice and Protection of Juveniles, a public advisory institution. The report cites several criminological and psychological papers besides three peer-reviewed neuroscientific publications (Paus et al., 2001; Adleman et al., 2002; Casey et al., 2005), which already were a bit dated at the moment of its publication (i.e., 2011). What were these neuroscientific studies about and what did they find?

Adleman et al. (2002) let subjects carry out the Stroop color word task, a classical psychological test for cognitive control, in an fMRI scanner. In this task, an impulsive reaction must be inhibited. The researchers compared three age groups, children (7–11 years, *N* = 8), adolescents (12–16 years, *N* = 11), and young adults (18–22 years, *N* = 11). They report that three areas in the prefrontal cortex were significantly more activated in the young adults than in the adolescents and that there was a similar pattern when comparing the young adults and the children; comparing the adolescents and the children did not yield such a result.

Much has been written about the statistical nature of such findings before and how this limits a direct translation to practical applications (Schleim and Roiser, 2009; Bigenwald and Chambon, 2019). But even as we take the findings as they are, there is a problem with respect to the legal justification: The new law introduces more possibilities to treat people in the age group 12 to 22 years *similarly*, but the fMRI scan yielded significant *differences*. In addition, the study cannot say anything about the upper threshold introduced in the new law because no subjects older than 22 were investigated. Finally, and more secondarily, that no differences were found between adolescents and children, whom the law treats differently, also does not support the legal age categories on the lower boundary.

The paper by Casey et al. (2005) is a review summarizing several studies on human brain development. They do not present much evidence in favor of specific age categories—and where they do, referring to a review by Thompson and Nelson (2001)—they say that the brain of a 6-year-old already amounts to 90% of the volume of an adult’s brain and that sensomotoric, associative, and prefrontal brain regions are almost completely developed by the age of 16. This obviously contradicts the reasoning of the legal justification, which emphasizes the continuous brain development until at least the age of 25, particularly of the prefrontal cortex. Casey and colleagues also emphasize the limitations of brain scans and caution against premature conclusions^8^.

The third and last neuroscientific paper quoted in support of the new legislation (Paus et al., 2001) is also a review paper and particularly discusses structural findings in people until the age of 30. The authors show two things: First, there seem to be developmental brain processes continuing until at least the age of 30; second, there are big inter-individual differences within each age group. The latter means that, for example, the *corpus callosum* connecting both hemispheres of a 6-year-old can be bigger than that of a 16-year-old. In support of the new legislation, one could say that the developmental processes seem to decrease the older one gets. However, these processes change gradually on a continuum and not in a categorical way, raising the question: How different would the *brain* processes or structures between two age groups have to be to justify a *legal* difference? This brings me to the next section, in which I will discuss some more general questions relevant to neurolaw.

## The Utility of Neurolaw

The previous section showed clearly that the neuroscientific evidence quoted in favor of the new adolescent criminal law in Netherlands does not support the new legal age thresholds. This does not leave these categories scientifically unjustified, but just shows that the *neuroscientific* studies presented by the Council and implicitly by the State Secretary do not match the intention of the lawmaker. The Council’s report quotes criminological and psychological evidence as well, but analyzing this goes beyond the purview of this paper. Morse (2006) coined the notion of a “brain overclaim syndrome,” which is now used by many who are criticizing exaggerated claims derived from neuroscience. The example analyzed in this paper is particularly odd in that the neuroscientific findings, on closer investigation, obviously contradict the claims in the legal justification. In the remainder of this section, I will present some thoughts on how a neuroscientifically justified law could be developed and what the prospects of such an endeavor are:

Most importantly, legal responsibility is not a neuroscientific category. Philosophers and forensic psychiatrists have analyzed before how different understandings of responsibility could be linked to the brain (Vincent, 2011, 2015; Meynen, 2014, 2016). A translation process between the domains must thus occur. This will necessarily involve an intermediary step of responsible *behavior*. Imagine that members of one age group generally show more irresponsible behavior than that of another age group. We may assume that this somehow relates to these groups’ developmental phases, including brain development. Whether an understanding of the neural processes underlying these behavioral differences is more informative than the already available developmental–psychological and behavioral evidence is an open question. A major contribution to the practical application in legal cases would be if neurotechnology allowed an estimation of someone’s legal responsibility in an *individual* case. For this, findings much stronger than the statistically significant differences between groups discussed in the previous section would have to be available. For the time being, we can have a look at psychiatry, where the lacking contribution of neuroscience to clinical practice has been discussed more critically in recent years (Frisch, 2016; Schleim, 2018, 2020; Gardner and Kleinman, 2019). Although there are thousands of neuroscientific studies reporting significant differences between patients and healthy controls or different groups of patients, not a single psychiatric disorder described in the common clinical manuals can reliably be diagnosed by a brain scan or another biological test.

Article 77c of the Dutch Criminal Code, as quoted previously, allows to apply the rules for juveniles when the “personality of the offender or the circumstances” of the criminal acts provide sufficient reason. Legal scholars found that public prosecutors, who can submit such a request to the court, do so rather on the basis of their intuition or pragmatic criteria such as whether the young offenders are still living with their parents, attending school, or having intellectual disabilities, and that judges seem to take their decisions in an inconsistent way:

“So far, unclarity and inconsistency remain regarding the applicable target group, type and severity of offences. […] The lack of legislative guidance has transformed the proposed flexible system, with ample discretion for the professionals involved, into a system in which uncertainty and inequality can prevail.” (Schmidt et al., 2020: 15).

Although the new Dutch adolescent criminal law is a real case of neurolaw in the sense that it is justified neuroscientifically, that is, it makes use of brain research on the *level of justification*, it does not provide any neuroscientific guidance on the *level of application*. I think that this is the law’s major flaw: It tells people, in particular public prosecutors and judges, something about brain development, but does not give them any tools to assess it. I am not denying the availability of such a tool in the future; all I am saying is that given the situation in psychiatry, for which there has been much more research with respect to clinical application in individual cases, such a possibility seems unlikely in the near future. If judges could see on a brain scan how criminally responsible an offender is—at the moment of committing the crime—that would be the real game changer for legal practice. In such a scenario, fixed age boundaries might be given up completely, enabling a tailor-made approach doing maximum justice to each individual.

## Conclusion

Scholars of different disciplines and countries discussed in much detail if and how courts made use of neuroscientific evidence (e.g., Schleim, 2011, 2012, 2019; Spranger, 2012; Farahany, 2015; Hoffman, 2018; Greely and Farahany, 2019). To my knowledge, neuroscience has never before influenced lawmaking in such a direct and explicit way as in the new Dutch adolescent criminal law. However, according to my analysis, the mismatch between the quoted neuroscientific evidence and the legal justification as well as the lack of practical applications call this initiative into question. A general problem is that the criminal law in most (if not all) countries uses fixed age thresholds to make *categorical* distinctions, whereas neuroscience finds differences of brain development on a *continuum*. This will make every fixed age look somewhat arbitrary from a scientific point of view^9^. Further questions related to other domains might be raised: If the age at which adolescents have to be held fully legally responsible is ever more increased, this might have implications for how we think about their capability to make contracts or consent to medical procedures as well (Meynen, 2016). Signing for a life insurance, for example, also implies a subject’s capacity for long-term planning. Should these ultimately also be evaluated on an individual basis? And which role does psychological and neuroscientific evidence play for these rules, presently and in the future? These are intriguing questions which must be addressed on another occasion, though.

## Data Availability Statement

The original contributions presented in the study are included in the article. Further inquiries can be directed to the corresponding author.

## Author Contributions

SS conceived and wrote the whole manuscript.

## Conflict of Interest

The author declares that the research was conducted in the absence of any commercial or financial relationships that could be construed as a potential conflict of interest.

## Footnotes

1 The literature sometimes speaks of 23 years as a maximum age, which was one of the proposals before passing the law. As the translation of the respective article of the Dutch Criminal Code provided below will make clear, though, the possibility is restricted to offenders *before reaching the age of 23*, i.e., up to a maximum of 22 years.

2 Translated from Dutch, Kamerstuk 28 741 Nr. 17 d.d. 25 juni 2011, p. 2.

3 Translated from Dutch, Kamerstuk 33 498 Nr. 3 d.d. 13 December 2012, pp. 12–13.

4 For those unfamiliar with the neurolaw literature, it is important to know that even fewer and less direct references to neuroscience/the brain in the past have sparked a hot debate on the legal relevance of neuroscience (Schleim, 2019).

5 Translated from Dutch, Advies “Het jeugdstrafproces: toekomstbestendig” d.d. 14 maart 2011, p. 18. Here I omitted the references, which will be discussed in the next section.

6 Translated from Dutch, article 77c, first paragraph.

7 Translated from Dutch, https://www.rechtspraak.nl/Organisatie-en-contact/Rechtsgebieden/Strafrecht/Paginas/Adolescentenstrafrecht.aspx (accessed on April 5, 2020).

8 In a newer review paper that was published after the Council’s report and in which Cohen and Casey (2014) explicitly discuss implications for adolescent criminal law, they summarize the study by Dreyfuss et al. (2014) who compared 13- to 17- with 18- to 27-year-olds in an experiment on impulsivity. The significant differences they report about these two groups also contradict the intention of the new law.

9 One reviewer raised the valid point that the brain differences between young adults until 22 years and older people need not be clear in all cases to inform the law, but that an 85% certainty would already be sufficient. I agree. But then we still have the problem that the study by Casey et al. (2005) reported that the development of sensomotoric, associative, and prefrontal brain regions is almost complete by the age of 16; and that Paus et al. (2001) found that brain development continues until at least the age of 30. We would thus still need an independent normative argument for how much difference in brain development is sufficient to justify a difference on the legal level, which goes beyond the scope of this paper.
